# Health-related behavioral changes following the use of psychedelics in naturalistic settings

**DOI:** 10.1016/j.pmedr.2025.103161

**Published:** 2025-06-30

**Authors:** Pedro J. Teixeira, Rakesh Jain, Andrew D. Penn, Steven P. Cole, Saundra Jain, Arlen C. Moller, Helena Amaro, Charles Raison

**Affiliations:** a CIPER - Faculty of Human Kinetics, University of Lisbon, Portugal; b Department of Psychiatry, Texas Tech University School of Medicine – Permian Basin, Midland, TX, United States; c School of Nursing, University of California, San Francisco, San Francisco, CA, United States; d Research Design Associates, Yorktown Heights, NY, United States; e School of Nursing, University of Texas at Austin, Austin, TX, United States; f Department of Psychology, Illinois Institute of Technology, Chicago, IL, United States; g Department of Psychiatry School of Medicine and Public Health, University of Wisconsin-Madison, Madison, United States

**Keywords:** Psychedelics, Health, Behavior, Substance use, Diet, impulsivity, naturalistic setting

## Abstract

**Objective:**

Psychedelics have been increasingly studied for their potential to influence mental health and well-being, yet their relationship with broader health behaviors remains underexplored. This study examined associations between lifetime psychedelic use and health-related behaviors, including substance use, dietary habits, and impulsive tendencies.

**Methods:**

Using an extensive cross-sectional online survey, we analyzed responses from 2510 US adults reporting at least one lifetime psychedelic experience. Participants retrospectively assessed changes in behaviors following psychedelic exposure.

**Results:**

Respondents reported improvements in various health behaviors, including reduced alcohol (66 %) and tobacco (49 %) use, improved dietary habits (49 %), and decreases in impulsive behaviors (48–72 %). Those who reported more frequent psychedelic use or engagement in microdosing were more likely to endorse positive behavioral changes (*p* < .001). Furthermore, while some participants reported harms associated with psychedelic use, the majority perceived lasting benefits.

**Conclusions:**

These findings suggest that psychedelic use is associated with broad behavioral adaptations beyond mental health, including important areas such diet, and alcohol and tobacco use. Compared with those who use full doses, participants who microdosed reported a more positive behavioral profile.

## 1 Introduction

Once stigmatized as substances of abuse, psychedelics are now recognized for their potential therapeutic benefits. Both classic (e.g., LSD, psilocybin) and non-classic psychedelics (e.g., ketamine, MDMA) have been associated with substantial and enduring improvements in psychiatric and behavioral outcomes. Psychedelic-assisted interventions have shown remarkable promise in treating tobacco and alcohol addiction, with studies reporting abstinence rates of tobacco use up to 67 % at twelve months after treatment (Johnson et al., 2017) or decreased alcohol use up to 32 weeks and significantly greater than controls (Bogenschutz et al., 2022).

The use of psychedelics in naturalistic settings has also been associated with cessation or reduction in alcohol use among individuals with prior alcohol use disorder (Garcia-Romeu et al., 2019) and reduction in risk of opioid dependence and abuse among individuals with a history of opioid misuse (Pisano et al., 2017). Other observational findings indicate that psychedelics may influence changes in health-related behaviors, such as eating and exercise habits (Teixeira et al., 2022) and are associated with reduced rates of heart disease and diabetes (Simonsson et al., 2021).

The potential of psychedelics to help regulate externalizing problems, like violent and criminal behaviors, has received limited attention despite its social relevance and some promising results. In a study by Kopra et al. (2023), individuals from the general population associated LSD or psilocybin use with positive changes across multiple domains, including depression, alcohol and drug use, but also frustration and anger. Hendricks et al. (2018) observed that lifetime use of classic psychedelics was associated with lower odds of past-year criminal behavior (e.g., theft, assault, violent offenses), whereas Jones and Nock (2022a) reported fewer past-year arrests among psychedelic users in the general population (e.g., property, violent offenses, substance-related crimes).

In sum, existing research offers support for the therapeutic benefits of classic and non-classic psychedelics on mental health and substance misuse, while emerging research suggests their impact may extend to broader health behaviors and impulse regulation. Critical gaps remain, however, in the understanding of psychedelics' behavioral impacts, and further studies are needed to confirm or refute whether the therapeutic effects observed in clinical contexts are generalizable to naturalistic settings. Naturalistic studies, and real-world evidence more broadly, are recognized as a valuable complement to clinical research, offering more ecological validity, the inclusion of larger and more diverse samples, and generating hypotheses to be tested in controlled settings (Carhart-Harris et al., 2022). It also circumvents the limitations of government funding and other practical barriers related to their Schedule I drug status (Barnett et al., 2025).

The Psychedelics and Wellness Study (PAWS) addresses some of these questions by examining the putative effects of naturalistic psychedelic use on a broad range of psychological and behavioral outcomes. In this large-scale survey study, participants reported significant reductions in depressive and anxious symptoms, alongside improvements in emotional well-being, as well as substantial reductions in suicidal ideation and aggressive impulses following psychedelic use (Raison et al., 2022).

The present study examined associations between lifetime psychedelic use and several health-related behaviors, namely eating habits, substance use (alcohol, cannabis, opiates, benzodiazepines), and impulses related to aggression, criminality, and suicidality, following psychedelic experiences. In addition, we analyzed differential association of changes with specific psychedelic substances, dosing, and frequency of use.

## 2 Methods

### 2.1 Study design

The study used an online platform to deliver a survey designed to assess participants' retrospective perspectives on the effects of their (past) psychedelic use. As entry criteria, they were asked to check a box for which classic psychedelic they had used Lysergic Acid Diethylamide (LSD), psilocybin, Dimethyltryptamine (DMT), 5-Methoxy-*N*,*N*-Dimethyltryptamine (5-MeO-DMT), ayahuasca, or mescaline (San Pedro or peyote) at least one time in their lifetime.

Participants first answered demographic and psychedelic usage questions, including preferred substance, lifetime use, and history of microdosing. They then completed several questionnaires, including two instruments specifically created for the PAWS study: the 26-item Psychedelic Change Questionnaire (PCQ-26) and the 8-item Negative Consequences Inventory (NCI-8) (Raison et al., 2022).

### 2.2 Participant recruitment and enrollment

A full description of the PAWS study has been presented elsewhere (Raison et al., 2022). Participants were recruited via online platforms, social media, word-of-mouth, in person, flyers/postcards, email, and snowball sampling. A full description of the sample characteristics is available in Supplementary Table. Eligibility required being 18 years or older and having used psychedelics at least once, and there were no other criteria required for enrollment. Eligible participants (*n* = 2510) accessed an online consent form detailing study's purpose, design, confidentiality, and risks/benefits. After consenting, they proceeded to the online survey. The PAWS study was conducted on an anonymous basis, between August of 2019 and July 2023, and participants were treated in accordance with ethical standards for research involving human participants as outlined in the Declaration of Helsinki. The Western Institutional Review Board (WIRB) determined the study to be exempt under 45 CFR § 46.104(d). All methods were carried out in accordance with relevant guidelines and regulations.

### 2.3 Instruments

The PCQ-26 queries emotional states that occur during the psychedelic experience (e.g., awe, connection, joy), symptoms common to different mental disorders (e.g., ruminative thinking, substance abuse) and behavioral changes attributed to psychedelic use. This study focused on item 25 – “As a result of your psychedelic experience(s), how would you rate the change in your eating habits?” – scored from 1 (very much improved) to 7 (very much worse).

The NCI-8 covers eight negative outcomes participants could ascribe to their psychedelic use – suicidal desire, criminal and aggressive impulses/behaviors; alcohol, cigarette, cannabis, benzodiazepine, and opiate/opioid misuse – scored from 1 (very much improved) to 7 (very much worse) (further details available in Raison et al., 2022).

Participants also indicated their preferred psychedelic substance (options included psilocybin, LSD, ayahuasca, DMT, ketamine, mescaline, and 5-MeO-DMT) and the number of times it was used. Additionally, participants indicated whether they microdosed psychedelics at some point in their lives.

### 2.4 Statistical analyses

A full description of the PAWS study has been presented elsewhere (Raison et al., 2022). Participants were recruited via online platforms, social media, word-of-mouth, in person, flyers/postcards, email, and snowball sampling. A full description of the sample characteristics is available in Supplementary Table. Eligibility required being 18 years or older and having used psychedelics at least once, and there were no other criteria required for enrollment. Eligible participants (*n* = 2510) accessed an online consent form detailing study's purpose, design, confidentiality, and risks/benefits. After consenting, they proceeded to the online survey. The PAWS study was conducted on an anonymous basis, and participants were treated in accordance with ethical standards for research involving human participants as outlined in the Declaration of Helsinki. The Western Institutional Review Board (WIRB) determined the study to be exempt under 45 CFR § 46.104(d). All methods were carried out in accordance with relevant guidelines and regulations.

## 3 Results

### 3.1 Changes in health behaviors

Overall, a large proportion of participants reported improvements in all behaviors, ranging from 35.9 % for cannabis use to 72.1 % for aggressive impulse control (see Table 1). More than half of respondents reported positive outcomes for alcohol misuse (66 %) and suicidal ideation (68.9 %). About half of the participants reported healthier eating behavior and positive effects in smoking (49.4 % and 48.5 %, respectively) as well as positive changes in criminal impulses (48.2 %). A substantial number of participants reported reduced use of cannabis, opiates, and benzodiazepines (35.9 %, 40.2 %, and 36.8 %, respectively). For all behaviors, those reporting positive outcomes largely chose “much” or “very much” improved, compared to “minimally” improved. The variability in sample size across variables matches the number of participants for whom the variable applies (e.g., if participants did not smoke before psychedelic use, they did not answer about change in that variable).

**Table 1:** Response frequency and percentage for changes in health behaviors associated with psychedelic usage (adults, online survey, August 2019 until July 2023).

	N^a^	Much/Very Much improved		Minimally improved		Total positive		No change		Negative change	
		n	%	n	%	n	%	n	%	n	%
Eating	2510	640	25.5	600	23.9	1240	49.4	1214	48.4	56	2.2
Cannabis misuse	1808	369	20.4	280	15.5	649	35.9	1007	55.7	152	8.4
Aggressive impulses	1782	847	47.5	437	24.5	1284	72.1	462	25.9	36	1.9
Desire to die/suicide	1711	942	55.1	236	13.8	1178	68.8	496	29.0	37	2.2
Alcohol misuse	1660	791	47.7	305	18.4	1096	66.0	510	30.7	54	3.2
Criminal impulses	1381	458	33.2	207	15.0	665	48.2	651	47.1	65	4.6
Cigarette smoking	1239	414	33.4	187	15.1	601	48.5	535	43.2	103	8.3
Benzodiazepine misuse	560	166	29.6	40	7.1	206	36.8	335	59.8	19	3.4
Opiate misuse	537	183	34.1	33	6.1	216	40.2	311	57.9	10	1.8

a Respondents for whom items applied to.

Regarding the relationship between behavior change and characteristics of psychedelic use (Table 2), participants reporting positive changes had a higher median lifetime use of psychedelics compared to the groups reporting no change or negative outcomes. Except for cannabis misuse, the difference between groups was significant across all behaviors. Compared to those who did not microdose, participants who included this practice reported higher rates of positive change, with significant differences across all health behavior domains. Finally, psilocybin users were more likely to report positive changes than LSD in eating behaviors, but all other comparisons were not significant.

**Table 2:** Associations between change in health behaviors and psychedelic use, microdosing, and type of psychedelic (adults, online survey, August 2019 until July 2023).

	Number of times a psychedelic was used^a^		Microdosedb		Psilocybin vs. LSDb	
	Positive Change (Median)	No/Negative Change (Median)		Positive Change (%)		Positive Change (%)
Eating behaviors	16.0	10.0	Yes	57.6	Psilocybin	51.7
			No	36.6	LSD	43.8
	<0.001		<0.001		<0.010	
Alcohol misuse	20.0	10.0	Yes	76.3	Psilocybin	67.8
			No	49.3	LSD	62.1
	<0.001		<0.001		0.080	
Cigarette smoking	20.0	12.0	Yes	56.0	Psilocybin	49.6
			No	36.1	LSD	43.8
	<0.001		<0.001		0.116	
Cannabis misuse	15.0	12.0	Yes	38.5	Psilocybin	36.1
			No	31.4	LSD	33.6
	0.079		<0.001		0.541	
Opiate misuse	26.5	10.0	Yes	50.9	Psilocybin	40.2
			No	24.7	LSD	35.5
	<0.001		<0.001		0.580	
Benzodiazepine misuse	25.0	10.0	Yes	46.2	Psilocybin	37.1
			No	21.8	LSD	32.0
	<0.001		<0.001		0.384	
Aggressive impulses	15.0	10.0	Yes	78.3	Psilocybin	71.6
			No	61.7	LSD	71.4
	<0.001		<0.001		0.514	
Criminal impulses	20.0	10.0	Yes	55.1	Psilocybin	49.8
			No	37.2	LSD	47.2
	<0.001		<0.001		0.256	
Desire to die by suicide	15.0	10.0	Yes	75.6	Psilocybin	68.6
			No	57.9	LSD	69.0
	<0.001		<0.001		0.871	

^a^Independent-Samples Kruskal-Wallis Test was used to compare positive and negative/no changes in health-related behaviors according to lifetime use of psychedelics (*p*-value reported).

bNon-Parametric Chi-square Test of Association was used to compare positive changes in health-related behaviors according to microdosing experience and type of psychedelic (p-value reported).

## 4 Discussion

The present study extends previous findings from the PAWS study (Raison et al., 2022) on the potential impact of naturalistic psychedelic use in health-related behaviors. Our results indicate that a significant proportion of individuals who have used psychedelics report positive changes in various health behaviors, with notable improvements in eating habits and alcohol, tobacco, and use of other substances. These findings strengthen prior research suggesting that psychedelics may catalyze positive behavioral modifications beyond mental health benefits (Neuhaus and Slavich, 2022; Teixeira et al., 2022) and further support the hypothesis that psychedelic users engage in health-protecting behaviors at a higher rate than non-users (Aday et al., 2024). Moreover, results align with two studies of ayahuasca users who have reported higher levels of physical activity, improved diet, and less alcohol consumption in comparison with the general population (Kohek et al., 2023; Ona et al., 2019).

Likewise, the reported reductions in impulsive behaviors, including aggression, criminal tendencies and addiction mirror prior findings demonstrating lower rates of violent and antisocial behaviors (Hendricks et al., 2018) as well as cocaine (Jones and Nock, 2022b) and opioid use (Pisano et al., 2017), among individuals with lifetime psychedelic use.

An interesting aspect of our results is the differential impact of psychedelic substances on behavioral change. While psilocybin was more strongly associated with positive changes in eating behaviors, no significant differences were observed between psilocybin and LSD for other behaviors. These findings suggest that while the general transformative potential of psychedelics is consistent across different substances, specific compounds may be associated with distinct health behaviors.

Another noteworthy observation is that participants who had engaged in microdosing were significantly more likely to report positive changes across multiple health domains compared to those who had not – a finding further supported by evidence suggesting that microdosing might be a tool for cognitive and emotional enhancement (Polito and Liknaitzky, 2022). Research on microdosing is still in its early stages and suggests that, in healthy adults, this practice is generally safe and may produce short-term behavioral and neural effects. However, these findings have not yet been extended to clinical populations as evidence for an effective mental health treatment (Murphy et al., 2023).

Despite the promising implications of our findings, several limitations should be acknowledged. First, the reliance on retrospective self-report data introduces potential biases (e.g., recall bias, social desirability). Second, our sample consisted of individuals who voluntarily participated in an online survey, likely leading to a selection bias favoring individuals with more positive psychedelic experiences. Third, even though the study identified associations between psychedelic use and behavior changes, causality cannot be inferred due to the cross-sectional design. Research employing longitudinal and controlled study designs is necessary to validate these findings and to elucidate the potential role of psychedelics in promoting healthier lifestyle behaviors.

In conclusion, our study builds on previous research by demonstrating that psychedelic use is associated with health-related behavior changes, including reduced substance misuse, healthier eating patterns, and diminished impulsive behaviors. These findings contribute to a growing recognition of psychedelics as potential catalysts of positive lifestyle changes that impact individual and societal well-being, while adding support to an emerging area of research and policy focused on the potential impact of psychedelics on public health at the population level (Kuiper et al., 2024).

## CRediT authorship contribution statement

**Pedro J. Teixeira:** Writing – original draft, Validation, Methodology, Conceptualization. **Rakesh Jain:** Writing – review & editing. **Andrew D. Penn:** Writing – review & editing. **Steven P. Cole:** Writing – review & editing, Methodology, Formal analysis. **Saundra Jain:** Writing – review & editing. **Arlen C. Moller:** Writing – review & editing. **Helena Amaro:** Writing – review & editing, Visualization. **Charles Raison:** Writing – review & editing, Data curation, Conceptualization.

## Declaration of generative AI and AI-assisted technologies in the writing process

During the preparation of this work, the authors used Open IA ChatGPT in order to review the writing style/format. After using this tool/service, the authors reviewed and edited the content as needed and take full responsibility for the content of the published article.

## Funding

This research did not receive any specific grant from funding agencies in the public, commercial, or not-for-profit sectors.

## Declaration of competing interest

The authors declare that they have no known competing financial interests or personal relationships that could have appeared to influence the work reported in this paper.

## Appendix A Supplementary data

Sample characteristcs (adults, online survey, July 2019 until July 2023) Image 1
