# Supplementary material for: Health-related behavioral changes following the use of psychedelics in naturalistic settings
Source: Prev Med Rep. 2025 Jun 30;56:103161. doi: 10.1016/j.pmedr.2025.103161 (PMC12271800; doi:10.1016/j.pmedr.2025.103161)
Supplement: Supplementary file 1 — Sample characteristcs (adults, online survey, July 2019 until July 2023) [file mmc1.docx]

**Supplementary Table.** Sample characteristics (adults, online survey, August 2019 until July 2023).

**Variables *N* % *M* *sd* Range**

Age (yrs.) 2,510 35.17 14.33 18 – 86

Gender

Female 1,222 48.7

Male 1,253 49.9

Other 35 1.4

Education

Less than high school 43 1.7

High school or equivalent 270 10.8

Some college 573 22.8

Associate degree 166 6.6

Bachelor’s degree 705 28.1

Graduate degree 479 19.1

Professional degree 274 10.9

Preferred psychedelic

Psilocybin (magic mushrooms) 1,295 51.6

LSD 755 30.1

Ayahuasca 145 5.8

DMT 80 3.2

Ketamine 69 2.7

Mescaline/peyote/San Pedro/other

mescaline containing cacti 51 2.0

Other designer/synthetic 46 1.8

5-MeO-DMT 42 1.7

Salvia 10 0.4

lboga/Ibogaine 8 0.3

2C-B 7 0.3

2C-E 2 0.1

Total number of times taken any

psychedelic in lifetime 2,510 38.55 76.15 1 – 500

Ever micro-dosed a psychedelic

substance

Yes 1,525 60.8

No 985 39.2

Healthcare provider

Yes 207 8.2

No 2,303 91.8

Based on Raison, C. L., Jain, R., Penn, A. D., Cole, S. P., & Jain, S. (2022). Effects of naturalistic psychedelic use on depression, anxiety, and well-being: Associations with patterns of use, reported harms, and transformative mental states. *Frontiers in Psychiatry*, *13*, 831092. <https://doi.org/10.3389/fpsyt.2022.831092>
